# Structures of alternatively spliced isoforms of human ketohexokinase

**DOI:** 10.1107/S0907444908041115

**Published:** 2009-02-20

**Authors:** Chi H. Trinh, Aruna Asipu, David T. Bonthron, Simon E. V. Phillips

**Affiliations:** a Astbury Centre for Structural Molecular Biology, Institute of Molecular and Cellular Biology, University of Leeds, Leeds LS2 9JT, England; b Leeds Institute of Molecular Medicine, University of Leeds, St James’s University Hospital, Beckett Street, Leeds LS9 7TF, England; c Research Complex at Harwell, Diamond Light Source Ltd, Diamond House, Chilton, Didcot OX11 0DE, England

**Keywords:** ketohexokinase, alternatively spliced isoforms, fructokinase, fructosuria

## Abstract

A molecular understanding of the unique aspects of dietary fructose metabolism may be the key to understanding and controlling the current epidemic of fructose-related obesity, diabetes and related adverse metabolic states in Western populations. Fructose catabolism is initiated by its phosphorylation to fructose 1-phosphate, which is performed by ketohexokinase (KHK). Here, the crystal structures of the two alternatively spliced isoforms of human ketohexokinase, hepatic KHK-C and the peripheral isoform KHK-A, and of the ternary complex of KHK-A with the substrate fructose and AMP-PNP are reported. The structure of the KHK-A ternary complex revealed an active site with both the substrate fructose and the ATP analogue in positions ready for phosphorylation following a reaction mechanism similar to that of the pfkB family of carbohydrate kinases. Hepatic KHK deficiency causes the benign disorder essential fructosuria. The effects of the disease-causing mutations (Gly40Arg and Ala43Thr) have been modelled in the context of the KHK structure.

## Abstract 2

The structures of the two alternatively spliced isoforms of human ketohexokinase, hepatic KHK-C and peripheral KHK-A, and of the ternary complex of KHK-A with the substrate fructose and AMP-PNP have been solved. The differences between KHK-A and KHK-C resulting from the spliced region are subtle and affect thermostability and probably flexibility; the mutations causing fructosuria were modelled.

## 1. Introduction   

Fructose is a major carbohydrate component of most human diets. However, the greatly increased fructose load in Western diets in recent decades has been implicated in the development of adverse metabolic states, namely glucose intolerance, hyperlipidaemia, obesity, insulin resistance, diabetes and gout (Johnson *et al.*, 2007 ▶). The primary site of dietary fructose metabolism is the liver. Ketohexokinase (fructokinase; KHK) is the initiating enzyme of fructose catabolism, converting fructose to fructose 1-phosphate. High levels of KHK are also present in the renal cortex and the small intestine and KHK is induced by fructose-rich diets (Koo *et al.*, 2008 ▶).

Hepatic fructokinase deficiency (caused by recessive mutations in the *KHK* gene) underlies essential fructosuria, a benign inborn error of metabolism. This is characterized by a large and persistent rise in blood fructose after the ingestion of fructose, sucrose or sorbitol and the excretion of 10–20% of the ingested load in the urine (Steinmann *et al.*, 2001 ▶). The only disease-causing mutations reported to date result in the amino-acid substitutions Gly40Arg and Ala43Thr, which are present in a compound heterozygous state in affected individuals in one family (Bonthron *et al.*, 1994 ▶).

Two KHK protein isoforms are encoded by alternatively spliced KHK mRNAs. These are referred to as KHK-C (predominantly central; hepatic/renal/intestinal) and KHK-A (more widely distributed but with a low expression level; Hayward & Bonthron, 1998 ▶). No physiological function is presently defined for KHK-A. Using recombinant proteins, we have previously shown that both KHK isoforms are enzymatically active; however, KHK-A is considerably more thermostable and has a poorer affinity for fructose than KHK-C (Asipu *et al.*, 2003 ▶). The Ala43Thr mutation retains bio­chemical activity but results in increased thermolability, with a disproportionate effect at physiological temperature for KHK-C compared with KHK-A. We have therefore hypothe­sized that the effect of Ala43Thr *in vivo* may be to confer a selective deficiency of KHK-C (hepatic/renal/intestinal), with peripheral KHK-A activity being preserved.

**Figure 1:**
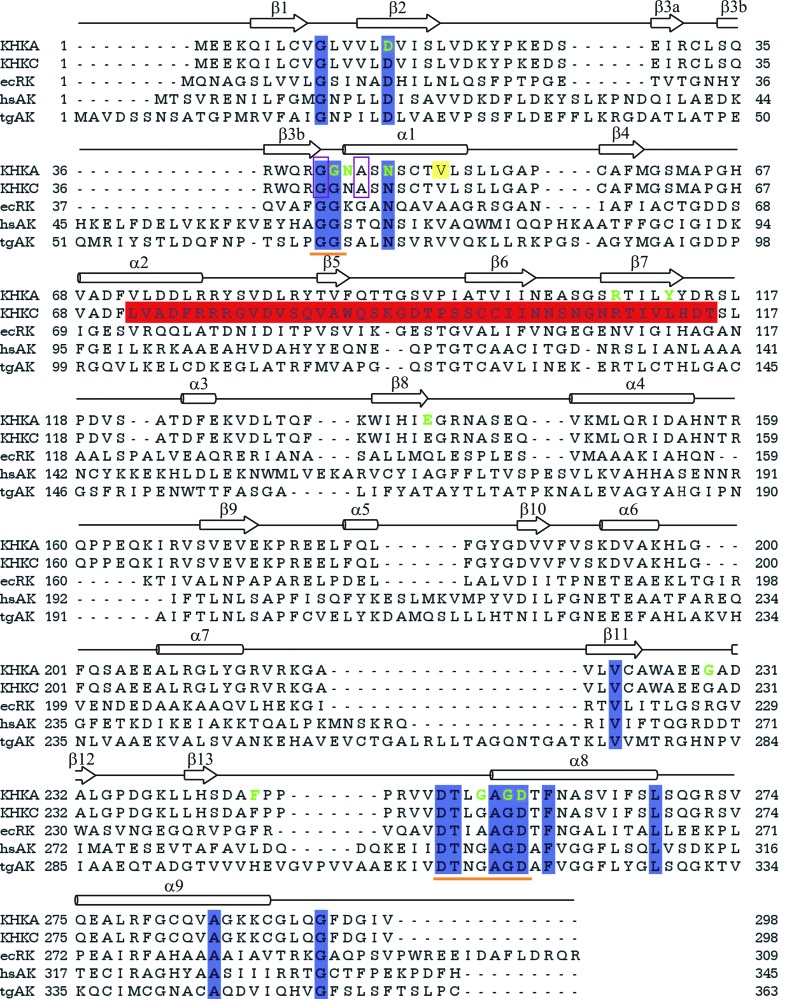
Sequence alignment of KHKs and related proteins based on observed secondary structure. Regions in which the secondary structures are conserved throughout all five members of the ribokinase family are shown above the sequences. The numbering system used to denote the secondary structure is based on that of KHK-A. The alternatively spliced region that differs between the KHK-A and KHK-C isoforms is highlighted in red in the KHK-C sequence. There are additional strands and helices in ecRK, hsAK and tgAk, but the overall topologies of the structures are similar to that of KHK. The residues that make interactions with the substrates are shown in green. The mutated residues Gly40 and Ala43 are boxed in purple and completely conserved residues are highlighted in blue. The highly conserved ‘GG’ and ‘DT*X*GAGD’ motifs are underlined in orange. Val49, which is coloured yellow, represents the site of a polymorphic variant (V/I) of KHK (Bonthron *et al.*, 1994 ▶). The sequences in the table are KHK-A, *Homo sapiens* ketohexokinase KHK-A (Swiss-Prot P50053); KHK-C, *H. sapiens* ketohexokinase KHK-C (Swiss-Prot P50053-2); ecRK, *E. coli* ribokinase (Swiss-Prot P0A9J6; PDB code 1rkd; Sigrell *et al.*, 1998 ▶); hsAK, *H. sapiens* adenosine kinase (Swiss-Prot P55263; PDB code 1bx4; Mathews *et al.*, 1998 ▶); tgAK, *Toxoplasma gondii* adenosine kinase (Swiss-Prot Q9TVW2; PDB code 1lii; Schumacher *et al.*, 2000 ▶). Sequence alignment was carried out using the program *Jalview* (Clamp *et al.*, 2004 ▶).

Ketohexokinase has no significant primary-structure similarity to other mammalian hexokinases, but analysis of the amino-acid sequence using *BLASTP* 2.2.18+ (Altschul *et al.*, 1997 ▶) indicates that KHK belongs to the pfkB family of carbohydrate kinases, which also includes adenosine kinase (Schumacher *et al.*, 2000 ▶; Mathews *et al.*, 1998 ▶), ribokinase (Sigrell *et al.*, 1998 ▶), 2-keto-3-deoxygluconate kinase (KDGK; Ohshima *et al.*, 2004 ▶) and aminoimidazole riboside kinase (AIRs kinase; Zhang *et al.*, 2004 ▶). All these enzymes are phosphotransferases with an alcohol group as the phosphate acceptor. The phylogenetic relationship between the pfkB proteins (which evolved by a combination of gene duplication and functional diversification) has been well established (Hansen *et al.*, 2007 ▶). Alternative splicing of the *KHK* gene selects either one or the other of two adjacent 135 bp exons that represent the evolutionary descendants of a paralogous local exon duplication (Hayward & Bonthron, 1998 ▶). The two resulting protein isoforms differ within a region of 44 residues (Fig. 1 ▶) that translates structurally to β-strand 5 of the central α/β-fold and β-strands 6 and 7 of the extended four-stranded β-sheet that forms the dimer interface.

Wu *et al.* (1991 ▶) and Bork *et al.* (1993 ▶) identified two short regions of strong sequence homology in members of the ribokinase family. One of these boxes is located near the N-­terminus and contains a conserved diglycine repeat, while the other is near the C-terminus and contains a conserved DT*X*GAGD motif (Fig. 1 ▶). Here, we report the structure of human KHK-A and of its complex with fructose and a nucleotide analogue and that of the alternatively spliced form KHK-C in the absence of ligands.

## 2. Experimental procedures   

### 2.1. Expression, purification and crystallization   

The expression in *Escherichia coli* and purification of the two isoforms of human ketohexokinase, KHK-C and KHK-A, were performed as described previously (Asipu *et al.*, 2003 ▶). The final purified protein fractions, which were eluted in buffer containing 120 m*M* Tris acetate pH 8.3 and 1 m*M* DTT, were concentrated to 10 mg ml^−1^ using a Centricon-30 concentrator (Amicon, Germany). Crystals of KHK-A and KHK-­C were grown at 289 K by the sitting-drop vapour-diffusion method. 2 µl protein solution was mixed with an equal volume of the appropriate precipitant solution and equilibrated against 1 ml of the same precipitant solution. KHK-A crystals were grown from a precipitant solution containing 0.5–0.9 *M* ammonium sulfate and 0.5 *M* lithium sulfate in 100 m*M* sodium citrate pH 5.4–6.0 and belonged to space group *I*222, with unit-cell parameters *a* = 61.3, *b* = 109.6, *c* = 146.3 Å. Crystals were also grown in the presence of 8 m*M* fructose or 2 m*M* xylulose, 10 m*M* AMP-PNP and 6 m*M* magnesium chloride. There is one monomer in the asymmetric unit, with the biological dimer being formed *via* a crystallo­graphic twofold axis.

KHK-C crystals were grown from a precipitant solution containing 18–22%(*w*/*v*) PEG 3000 in 100 m*M* sodium acetate pH 4.0–4.5 and belonged to space group *P*2_1_2_1_2_1_, with unit-cell parameters *a* = 90.6, *b* = 140.7, *c* = 179.3 Å. There are two dimers per asymmetric unit (subunits *A* and *B* and subunits *C* and *D*).

### 2.2. Data collection, structure determination and refinement   

X-ray diffraction data for the native enzymes and the complex were collected at 100 K on stations 14.1 and 14.2 at Daresbury Synchrotron Radiation Source using an ADSC Quantum IV charge-coupled device (CCD) detector. For the KHK-A crystals 25%(*v*/*v*) glycerol was added to the crystallization buffer as a cryoprotectant. The KHK-C crystals were less robust and cracked during serial addition of glycerol. As a result, the KHK-C crystals were instead transferred to 25% 2-­methyl-2,4-pentanediol (MPD) before data collection. All native and derivative X-ray diffraction data sets were integrated using *MOSFLM* (Leslie, 1992 ▶) and scaled and reduced using *SCALA* and *TRUNCATE* from the *CCP*4 program suite (Collaborative Computational Project, Number 4, 1994 ▶). Data-processing statistics are shown in Table 1 ▶.

Initial attempts to solve the structure of KHK-A using molecular replacement using the structure of ribokinase (Sigrell *et al.*, 1998 ▶) as a search model were unsuccessful. The KHK-A structure was therefore determined by the single isomorphous replacement (SIR) method. After screening a wide range of heavy-atom compounds, a useful derivative of KHK-A was obtained by soaking a crystal in mother liquor containing 1 m*M* potassium hexachloroplatinate(IV) (K_2_PtCl_6_) for 10 min. Derivative data were collected at 100 K to a resolution of 2.8 Å using a Rigaku RU-H3R generator with an R-AXIS IV^++^ detector. The heavy-atom derivative data were scaled to the native data using *SCALEIT* from the *CCP*4 program suite (Collaborative Computational Project, Number 4, 1994 ▶). One heavy-atom site was found by Patterson methods using *SOLVE* (Terwilliger & Berendzen, 1999 ▶) with data in the 10.0–3.0 Å resolution range. The quality of the initial electron-density map was subsequently improved using *RESOLVE* (Terwilliger, 2000 ▶). The density-modified 3.0 Å SIR map was noisy, but at 6.0 Å it was clear and the structure of *E. coli* ribokinase complexed with ribose and ADP (PDB code 1rkd) could readily be positioned into the density, additionally determining the correct enantiomorph. Rigid-body refinement using *CNS* (Brünger *et al.*, 1998 ▶) was carried out on the ribokinase model (Sigrell *et al.*, 1998 ▶) to produce a starting model for KHK-A.

The model was built using *O* (Jones *et al.*, 1991 ▶) and refined first using *CNS* (Brünger *et al.*, 1998 ▶) and then using *REFMAC*5 (Murshudov *et al.*, 1997 ▶) with data to 1.86 Å resolution. The progress of the refinement was monitored by means of the free *R* factor (Brünger, 1992 ▶). Water molecules were included in the model when the *R* factor reached 30%, but only where clear peaks were present in both the 2*F*_o_ − *F*_c_ and *F*_o_ − *F*_c_ maps and where appropriate hydrogen bonds could be made to surrounding residues or to other water molecules. Refinement was judged to be complete when the *R* factor had converged and no significant interpretable features remained in the *F*_o_ − *F*_c_ map. The geometry of all the models was monitored using the program *PROCHECK* (Laskowski *et al.*, 1993 ▶). A summary of the refinement statistics is given in Table 1 ▶.

The structure of KHK-A complexed with fructose and AMP-PNP was determined by first transferring the crystals of KHK-A with fructose and AMP-PNP grown using ammonium sulfate into a buffer containing 100 m*M* Tris–HCl pH 8.5, 10% PEG 400, 10 m*M* AMP-PNP, 8 m*M* fructose and 6 m*M* Mg^2+^ with an increasing percentage of glycerol up to 25%(*v*/*v*) prior to flash-cooling in liquid nitrogen. The crystal structure of KHK-C was determined by molecular replacement using a subunit of KHK-A as the search model with *AMoRe* (Navaza, 1994 ▶), since no solutions could be obtained using a KHK-A dimer as a search model. The structure was refined using the same procedure as used for KHK-A.

### 2.3. Modelling   

To model the structure of KHK-C bound to fructose and AMP-PNP, the residues in the alternately spliced region of the structure of KHK-A bound to fructose and AMP-PNP were mutated to match those of KHK-C using *Coot* (Emsley & Cowtan, 2004 ▶) and the rotamers for the side chains of the mutated residues were manually selected to minimize steric repulsion with neighbouring atoms. Four structures were used for energy-minimization modelling: KHK-A, the KHK-A ternary complex, KHK-C (dimer *AB*) and the KHK-A ternary complex with a mutated splice region. Energy-minimization modelling was carried out using *AMBER* (Pearlman *et al.*, 1995 ▶) with the force field of Cornell *et al.* (1995 ▶). The structures were individually processed using the *XleaP* module of *AMBER* and H atoms were added to the system. Modelling of KHK fructosuria-causing mutations was performed in a similar manner.

## 3. Results   

### 3.1. Structure determination   

Three crystal structures of human ketohexokinase were determined: those of KHK-A, of KHK-A in complex with fructose and adenyl-5′-yl imidodiphosphate (AMP-PNP) and of KHK-C.

The structure of KHK-A under high-salt conditions was determined to 1.86 Å resolution from crystals grown in the absence of additional substrates using a combination of low-resolution single isomorphous replacement with a potassium hexachloroplatinate(IV) (K_2_PtCl_6_) derivative and molecular replacement with *E. coli* ribokinase (Sigrell *et al.*, 1998 ▶; PDB code 1rka). The final structure revealed the presence of a monomer in the asymmetric unit, with the biological dimer being formed *via* a crystallographic twofold axis. Good electron density was present for residues 3–298 and included all side chains apart from those of Glu3 and Glu163, which were truncated to alanines. The final unliganded KHK-A model contained 296 residues, 269 water molecules and three sulfate ions and had a crystallographic *R* factor of 20.5% and an *R*_free_ of 23.4% (Table 1 ▶). Residual weak electron density in the nucleotide site suggested partial occupancy of nucleotide carried over in protein purification and one of the bound sulfate ions lies in the middle of the active site. Structure determination of KHK-A cocrystallized with AMP-PNP and with AMP-PNP and fructose grown from similar ammonium sulfate conditions yielded similar maps to that of unliganded KHK-A (data not shown). A sulfate ion binds at the expected site of the γ-phosphate of AMP-PNP, probably impeding the binding of cofactor and substrate.

The structure of the ternary complex of KHK-A with fructose and AMP-PNP was also determined to 2.1 Å resolution. By transferring a crystal to Tris buffer containing additional fructose, the substrate and nucleotide were built into good density. The final model of the KHK-A complex contains 296 residues (residues 3–298), 248 water molecules, one sulfate ion, one glycerol molecule, one fructose molecule and one molecule of AMP-PNP. The structure was refined to *R* = 19.3% and *R*_free_ = 22.8% (Table 1 ▶).

The crystal structure of the alternatively spliced form KHK-­C was determined to 2.9 Å resolution by molecular replacement using the KHK-A structure as a search model. KHK-C crystallized in space group *P*2_1_2_1_2_1_, with unit-cell parameters *a* = 90.6, *b* = 140.7, *c* = 179.3 Å and two dimers per asymmetric unit (subunits *A* and *B* and subunits *C* and *D*). The quality of the electron-density map was much poorer than for KHK-A and for the *CD* dimer compared with the *AB* dimer. The final KHK-C model contains 1184 residues, with each subunit consisting of residues 3–298. 44 residues from all four subunits are modelled as alanines owing to a lack of side-chain electron density. 18 water molecules were added and the structure was refined to *R* = 23.6% and *R*_free_ = 28.1% (Table 1 ▶).

### 3.2. Overall structure   

**Figure 2:**
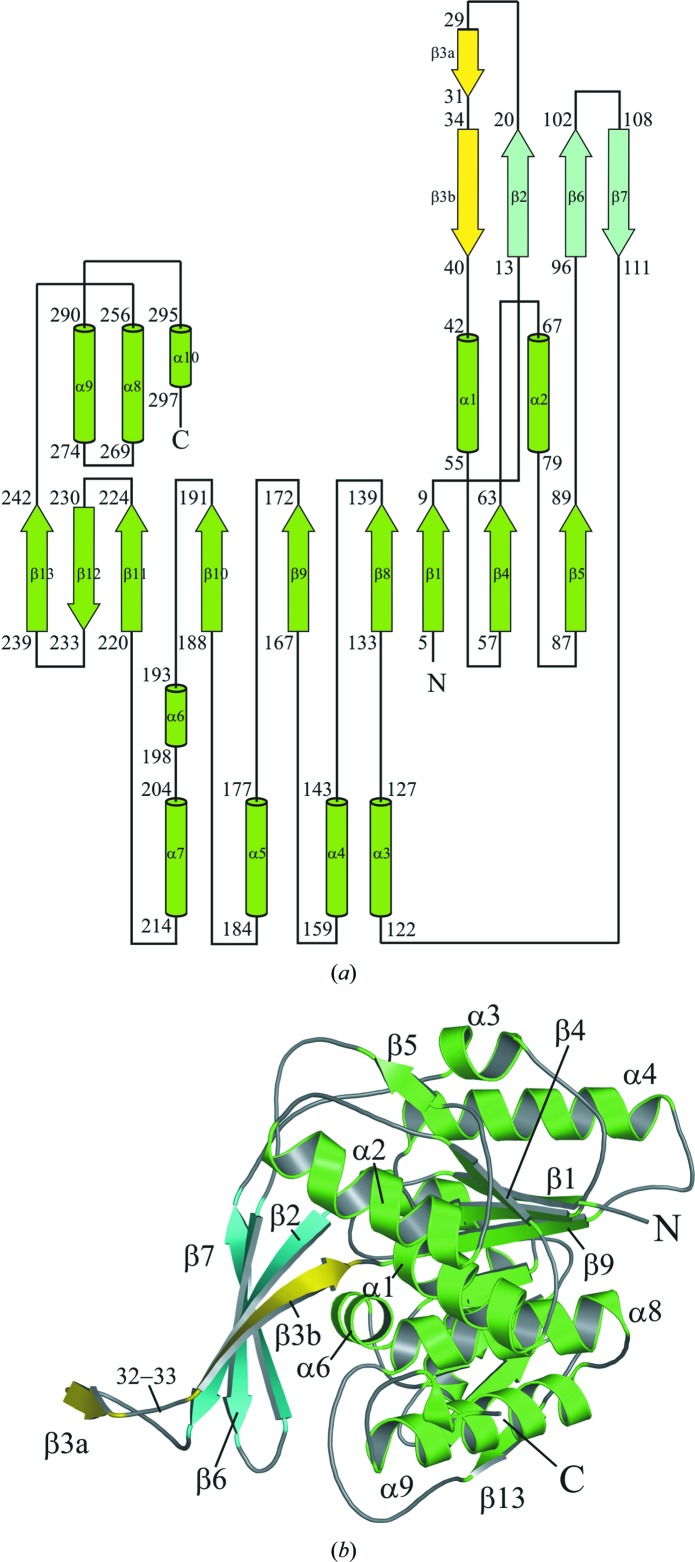
Structure of human KHK-A. (*a*) A topology diagram of the human ketohexokinase subunit. The α-helices are shown as cylinders and the β-­strands as arrows. Each secondary-structural element is labelled with its starting and ending sequence number. The secondary-structural elements were defined using *DSSP* (Kabsch & Sander, 1983 ▶). (*b*) A ribbon diagram representing the overall fold of the KHK subunit. The core domain of the monomer is comprised of a nine-stranded β-sheet flanked on both sides by α-helices. The four-stranded β-sheet formed by β2, β6, β7 (cyan) and β3 (yellow) extends away from the core domain, leaving a cleft for the active site. β3 is separated into two strands, β3a and β3b, by a bend at residues 32–33 that lies at the C-terminal end of β-strand β3a.

The KHK-A subunit has two distinct domains: a central α/β-fold and a four-stranded β-sheet. The α/β-fold consists of a nine-stranded β-sheet flanked on each side by five α-helices: α1, α2, α8, α9, α10 and α3, α4, α5, α6, α7 (Fig. 2 ▶). The overall structures of KHK-A and of its binary and ternary complexes are very similar, with no significant differences in main-chain conformation, except for a small region between residues 113 and 116 (Tyr113, Asp114, Arg115 and Ser116). The electron density around residues 115 and 116 is weaker, suggesting some flexibility, and alternative main-chain conformations may exist.

**Figure 3:**
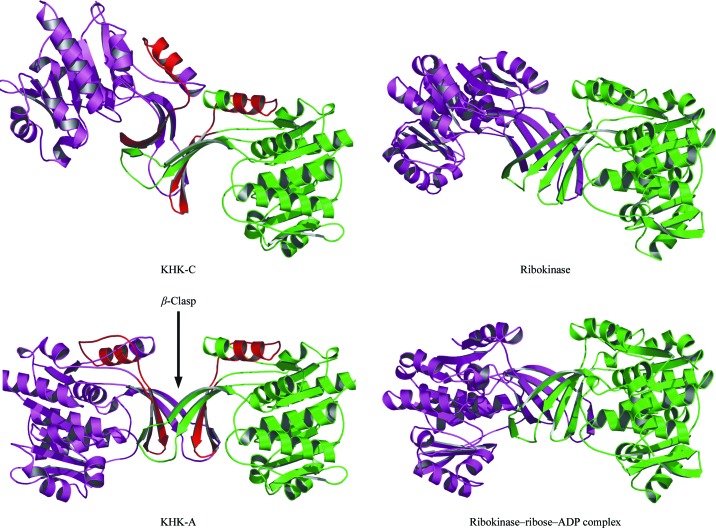
Comparison between human KHK-A and *E. coli* ribokinase. Ribbon diagrams are shown of KHK-A, of KHK-C and of *E. coli* ribokinase in the presence (PDB code 1rkd) and absence (PDB code 1rka) of ribose and ADP. The green subunit is shown in the same orientation in all structures. The alternative splicing of the *KHK* gene results in a different sequence for a single region of the chain between the two isoforms (residues 72–115), which is shown in red. These figures were generated using *PyMOL* (DeLano, 2002 ▶).

Gel filtration and nondenaturing polyacrylamide gel electrophoresis (Bais *et al.*, 1985 ▶; Raushel & Cleland, 1977 ▶) have demonstrated that KHK is a dimer in solution. In the crystal structure KHK-A is an elongated dimer with appropriate dimensions of 44 × 101 × 47 Å. The dimer interface is formed by the extended four-stranded β-sheets (β2, β3b, β6 and β7), which pack approximately orthogonally to form a distorted barrel (Fig. 3 ▶). The conformations of Cys32 and Leu33 disrupt β3, generating a sharp bend that separates it into two parts, β3a and β3b (Fig. 2 ▶). Alignment of various ketohexokinase sequences using *ClustalW*2 (Larkin *et al.*, 2007 ▶) show that cysteine and leucine residues are highly conserved at these positions throughout evolution (data not shown). β3a interacts with the four-stranded β-sheet from the other subunit, adding a fifth strand to the sheet in an arrangement described in *E. coli* ribokinase as a β-clasp structure (Sigrell *et al.*, 1998 ▶; Fig. 3 ▶). The KHK-A ternary-complex structure reveals the presence of one active site per subunit; it is located in a cleft between the α/β-domain and the four-stranded β-­sheet that forms the dimer interface such that the β-sheet forms a lid over the active site. The alternatively spliced region contains 44 residues that begin in the middle of α2 and extend through β5 of the central α/β-fold to β6 and β7 of the extended four-stranded β-sheet that forms the dimer interface (Fig. 3 ▶). Superposition of all C^α^ atoms for the KHK-A and the ternary-complex dimer structures gives a root-mean-square-deviation (r.m.s.d.) of 0.5 Å.

The overall structure of the KHK-C subunit is similar to that of the KHK-A subunit. There is no significant electron density in the active-site cleft, suggesting that no cofactor or substrate is bound, but the resolution is lower than for KHK-­A. Superposition of the corresponding individual structural domains (the central α/β-fold or the extended four-stranded β-­sheet) between KHK-A and KHK-C shows little conformational difference in their main chains. The central α/β-­domain of a single subunit of KHK-A superimposes onto subunits *A* or *B* of KHK-C with 0.5 Å r.m.s.d. for C^α^ atoms. Similarly, the extended four-stranded β-sheet of KHK-A superimposes onto subunits *A* and *B* of KHK-C with r.m.s.d.s of 0.9 and 0.4 Å, respectively. However, there is a notable difference between KHK-A and KHK-C in the relative orientation of these two structural domains. When the central α/β-fold regions of KHK-A and KHK-C are superimposed, the relative positions of the extended four-stranded β-sheets differ significantly between the two structures, with corresponding main-chain C^α^ atoms in this region deviating by up to 8 Å and side-chain atoms for some residues differing by up to 15 Å (see supplementary figure^1^). Since the dimer interface is formed by the β-clasp domains, these differences lead to a dramatic difference in the relative positions of the subunits in the two isoforms (Fig. 3 ▶). Approximately 1400 Å^2^ of accessible surface area is buried in the dimer interface for KHK-A, rising to approximately 1550 Å^2^ for KHK-C. The residues involved in the dimer interface remain very similar for the two isoforms.

### 3.3. Binding sites for fructose, AMP-PNP and magnesium ions   

**Figure 4:**
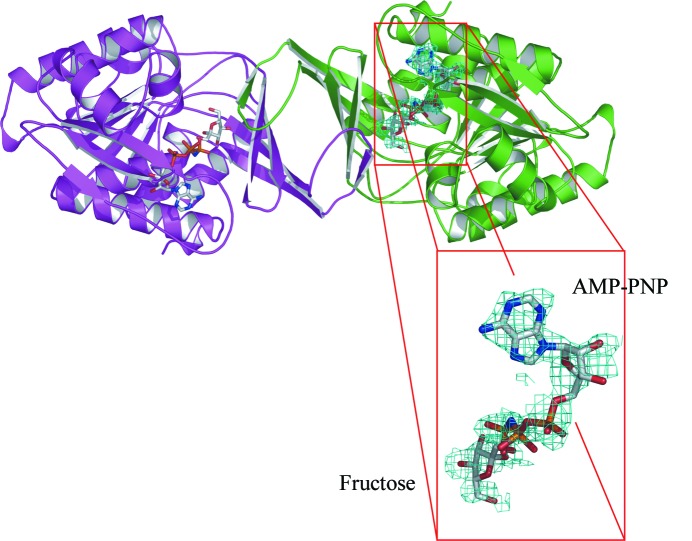
Electron density for bound fructose and AMP-PNP. A ribbon representation is shown of KHK-A complexed with fructose and AMP-PNP; the OMIT electron density around the fructose and AMP-PNP is contoured at the 1.0 r.m.s. level. This figure was generated with *PyMOL* (DeLano, 2002 ▶).

**Figure 5:**
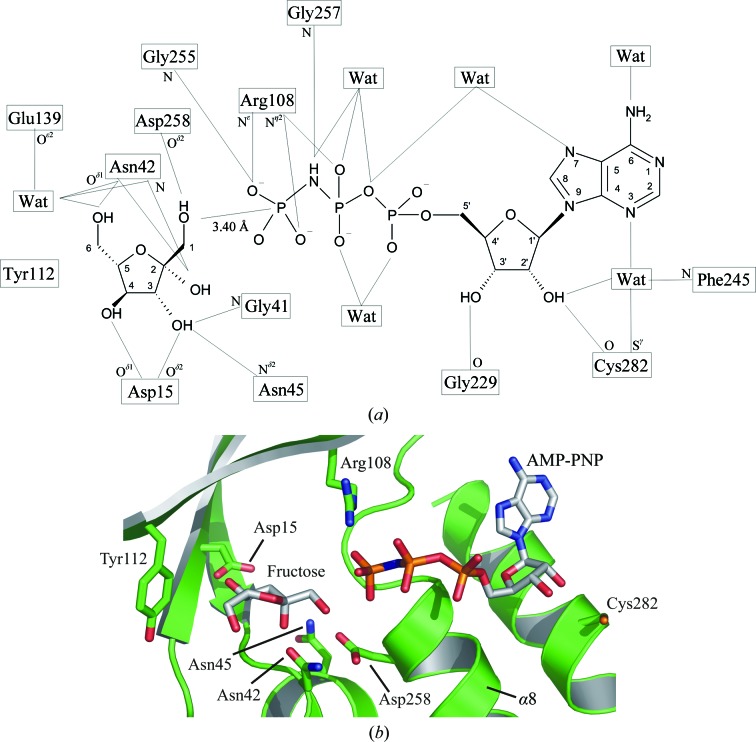
Substrate and cofactor interactions in the active site. (*a*) Schematic diagram of the KHK-A active site; thin lines show the hydrogen-bond interactions (≤3.2 Å) between the substrate and cofactor and the surrounding protein residues and water molecules. This figure was generated using *ISIS*/*Draw* 2.2.1 (MDL Information Systems). (*b*) Ribbon diagram showing the active site, with the residues interacting with the fructose and AMP-PNP shown in stick representation.

The AMP-PNP-binding site is located at one end of the active-site cleft, whilst fructose is bound at the other end, close to the four-stranded β-sheet (Fig. 4 ▶). In the structure of the ternary complex with fructose and AMP-PNP, the fructose molecule is almost completely buried, with only 5% of its solvent-accessible surface area exposed. The side chain of Tyr112 is orientated such that it closes the end of the binding cleft behind the C6 atom of fructose (Fig. 5 ▶). All five hydroxyl groups of fructose form direct hydrogen bonds to protein residues (Asp15, Gly41, Asn42, Asn45 and Asp258) *via* both main-chain and side-chain atoms; the bond between O6 and Asn42 O^γ1^ is quite long (3.3 Å) but has good geometry. O6 makes an additional indirect hydrogen bond to Glu139 O^∊2^ *via* a water molecule. The base and the ribose moieties of AMP-PNP are bound at the opposite end of the cleft where the site is much more open and a number of water molecules are present. The hydrophobic faces of the base are packed closely between the main chain at Ala226/Glu227 (the loop region between β11 and β12) and the side chain of Cys289, while C2 packs against Phe245/Pro246 (the loop region between β13 and α8). Ala226 adopts unfavourable ϕ and ψ angles of 49° and −124°, respectively, such that its carbonyl group lies parallel to the hydrophobic face of the base. All these residues are located at one end of the central β-sheet. The ribose hydroxyl groups make weak hydrogen bonds to Cys282 S^γ^ and Gly229 O, respectively. N6 and N7 of the base make hydrogen bonds to water molecules, while N3 hydrogen bonds *via* a buried water molecule to the 2′-OH of the ribose, Cys282 O and Phe245 N. The α-phosphate and β-phosphate groups are surrounded by water molecules, but the γ-phosphate is tightly bound to the GAGD motif (residues 255–258) at the N-­terminus of α8. The NH group linking the β- and γ-phosphates makes a tight contact with Gly257 N, which would be a hydrogen bond for ATP. The O1 atom of fructose lies only 3.4 Å from the P atom of the γ-phosphate, with an O1⋯P—NH angle of 152°.

## 4. Discussion   

A comparison of the structures of KHK-A and the ternary complex did not reveal any significant change in overall conformation. The two structures showed few detailed structural differences, except for a short section of the loop between β7 and α3 (113–116). These residues are located at the junction between the central α/β-fold and a four-stranded β-sheet domain. Analysis of domain motion using *DynDom* (Hayward *et al.*, 1997 ▶) showed no evidence of hinge motion. An independently determined structure of KHK-A in the absence of specific ligand has been deposited in the PDB (PDB code 2hlz; W. M. Rabeh, W. Tempel, L. Nedyalkova, R. Landry, C. H. Arrowsmith, A. M. Edwards, M. Sundstrom, J. Weigelt, A. Bochkarev & H. Park, unpublished work). Comparison with KHK-A using *DynDom* shows a small rotation between domains corresponding to a hinge motion that slightly opens the active site.

Comparison of the KHK-A ternary complex with chain *A* of KHK-C using *DynDom* reveals a large rotation between the two domains of the central α/β-fold and the four-stranded β-­sheet of 39.1°, again corresponding to a hinge motion opening the active site. Comparison of the KHK-A complex with chains *B*, *C* and *D* of KHK-C gives rotation results of 0°, 13.2° and 39.8°, respectively. The KHK-C dimers are therefore asymmetric, with one subunit in an ‘open’ conformation (*A* or *D*) and the other in a ‘closed’ conformation (subunits *B* or *C*). Modelling of a KHK-C dimer with both subunits in the open conformation generated a very close contact between opposing α2 helices, suggesting that only one subunit is likely to be open in any one molecule. The differences in conformation between KHK-A and KHK-C are unlikely to be a consequence of crystal packing, since the independent KHK-C molecules are in different environments but both have one subunit in the open conformation.

Superposition of all 44 C^α^ atoms of the splice region between KHK-A and KHK-C gives an r.m.s.d. of 1.8 Å. α2 and β5 superimpose well whilst β6 and β7 do not, with the largest displacement being 3.8 Å between the C^α^ atoms of residue 105 at the β-hairpin loop region between β6 and β7. Modelling the mutations of the splice-region residues of the KHK-A structure to those of KHK-C suggested that these mutated residues can be accommodated in the KHK-A conformation without any steric clashes, apart from residue Tyr112 in KHK-A and Leu112 in KHK-C. Replacing Leu112 in the KHK-C with tyrosine would cause a steric clash with Asp114. Localized rearrangement of the surrounding residues would be required to alleviate this. In KHK-A this is avoided by the main chain being re-orientated such that the side chains of Tyr112 and Asp114 are not close together.

In the ternary complex of KHK-A the side chain of Tyr112 has two alternative conformations: one with χ_1_ = 180° such that it closes one end of the binding cleft behind the fructose (Fig. 5 ▶) and another, with lesser occupancy as determined from the electron density, with χ_1_ = 60°, in which the side chain is orientated towards the solvent and so does not close off the binding cleft. In the high-salt KHK-A in the absence of substrate, the side chain of Tyr112 is fully orientated out into the solvent. It may play a role in allowing the entry of fructose, perhaps followed by closing the end of the cleft prior to phosphoryl transfer. In the closed conformation, Tyr112 makes a hydrophobic contact with the fructose substrate. In KHK-C, however, it is replaced by Leu112 and this change is probably the cause of the observed change in the *K*_m_ for fructose, which falls from 7 m*M* for KHK-A to 0.8 m*M* for KHK-C (Asipu *et al.*, 2003 ▶). A similar type of mechanism has been suggested for the reduced affinity of *Thermus thermophilus* KDGK for its substrate KG (Ohshima *et al.*, 2004 ▶). A tyrosine residue in the active-site/lid region has been suggested to mediate the opening and closing of the active site after substrate entry in the case of AIRs kinase (Zhang *et al.*, 2004 ▶).

Ketohexokinase is a member of the ribokinase family of sugar kinases (Bork *et al.*, 1993 ▶). Sequence and structural comparison between different members of the family has shown that KHK-A superimposes closely on representative members of the ribo­kinase family: *E. coli* ribokinase (RK; Sigrell *et al.*, 1998 ▶), *T. thermophilus* 2-­keto-3-deoxygluconate kinase (Oh­shima *et al.*, 2004 ▶), *Salmonella enterica* aminoimidazole riboside kinase (AIR; Zhang *et al.*, 2004 ▶), human adenosine kinase (Mathews *et al.*, 1998 ▶), human pyridoxal kinase (Cao *et al.*, 2006 ▶), *Pyrococcus furiosus* glucokinase (Ito *et al.*, 2003 ▶) and *Toxoplasma gondii* adenosine kinase (TdAK; Schumacher *et al.*, 2000 ▶). Superposition of 129 C^α^ atoms for the central α/β-fold between KHK-A and these structures from the ribokinase family results in r.m.s.d. values in the range 1.9–2.4 Å. The comparison showed that KHK-A is most similar to *E. coli* ribokinase and least similar to human pyridoxal kinase. The superimposed structures of the various members of the family revealed that the sugar and ATP-binding sites are very similar. The KHK-A and KHK-C sequences have approximately 19% identity to that of RK. In particular, sequence alignment (Fig. 1 ▶) shows KHK to have a common ancestry with *E. coli* ribokinase and a comparison of their overall structures is consistent with this, with highly conserved active sites. The substrate sugars and nucleotide ana­logues occupy the same locations within the active sites of their respective structures. The structure of *E. coli* ribokinase shows substantial conformational changes on binding sub­strate, with the lid formed by the β-sheet clasp region being more open in the apo form and closing down over the cleft on substrate binding (Sigrell *et al.*, 1999 ▶) to generate the active conformation. Similar observations have also been reported for several other members of the ribokinase family: TdAK (Schumacher *et al.*, 2000 ▶), TlGK (Ito *et al.*, 2001 ▶), PdxY (Safo *et al.*, 2004 ▶) and HMPP (Cheng *et al.*, 2002 ▶). In contrast, the structures of apo KHK-A and the KHK-A ternary complex did not exhibit a similar closing down over the active-site cleft of the β-sheet clasp region upon substrate binding and the KHK-A and KHK-A ternary-complex structures are very similar without any hinge motion as defined by *DynDom* (Hayward *et al.*, 1997 ▶). This suggests that the structure of apo KHK-A is either in the closed form as a result of crystal packing or that KHK-A does not undergo a conformational change upon substrate binding. Indeed, the structures of the *S. enterica* aminoimidazole riboside kinase with and without substrate have been shown not to undergo any conformational changes and the lid formed by the four-stranded β-sheet has been shown not to change in the presence of substrate and ATP (Zhang *et al.*, 2004 ▶).

AMP-PNP is resistant to hydrolysis, so that the KHK-A ternary-complex structure shows the protein in an active conformation poised for phosphoryl transfer. A search of the PDB for ATP-binding sites in other proteins using the program *SitesBase* (Gold & Jackson, 2006 ▶) revealed that the site in KHK-A is typical of ATP-binding pockets. There are few direct hydrogen bonds between the protein and the base, ribose or α- and β-phosphates, with most interactions being mediated by water molecules; this probably contributes to the relatively poor quality of the electron density even at 2.1 Å resolution.

The γ-phosphate, on the other hand, is tightly bound to the GAGD motif (255–258) at the N-­terminus of α8, with three direct hydrogen bonds and a fourth predicted for ATP to Gly257 NH. This forms the anion hole required to stabilize the transition state for phosphoryl transfer (Zhang *et al.*, 2004 ▶, 2006 ▶). The tight binding probably accounts for the stronger electron density observed for the γ-phosphate compared with the α- and β-phosphates, but a contribution from residual bound sulfate ion incompletely removed by crystal soaking cannot be completely ruled out. O1 of fructose lies only 3.4 Å from the γ-phosphate; it is almost collinear with the γP—NH bond and 2.7 Å from Asp258 O^δ2^. A catalytic mechanism has been pro­posed for ribokinase (Sigrell *et al.*, 1999 ▶) that is consistent with Asp258 functioning as a base, abstracting a proton from the O1 hydroxyl of fructose; this is followed by in-line nucleophilic attack by O1 at the γ-phosphoryl group of ATP, yielding fructose 1-phosphate as the product. The GAGD motif has been shown to be highly conserved (Wu *et al.*, 1991 ▶; Bork *et al.*, 1993 ▶) and supports the importance of Asp258 in the phosphorylation reaction.

Magnesium is essential for activity, but no clear evidence for any magnesium ions could be seen in the electron-density maps near the β- and γ-phosphates despite the addition of 6 m*M* MgCl_2_ to the crystallization solution. Similarly, Mg^2+^ was not observed for several other kinases which require Mg^2+^ for their activity (Cheng *et al.*, 2002 ▶; Ito *et al.*, 2003 ▶). In the *Tox. gondii* adenosine kinase structure the magnesium ion is located between the α- and β-phosphates (Schumacher *et al.*, 2000 ▶). Electron density that could correspond to either a water molecule or a magnesium ion with octahedral coordination is observed in the KHK-A ternary complex, but with slightly longer than expected distances for the apical coordinate waters at 3.4 and 3.7 Å. However, the quality of the density and the geometry do not allow unequivocal assignment of this density as a magnesium ion.

We have previously reported the biochemical effects of two KHK mutations (Gly40Arg and Ala43Thr) for which the affected members of a family with essential fructosuria are compound heterozygotes (Asipu *et al.*, 2003 ▶). A deficiency of hepatic fructokinase (KHK-C) was shown in these patients by ^31^P NMR studies of metabolites (Boesiger *et al.*, 1994 ▶). We have now modelled the effect of these mutations in the context of the KHK-A crystal structure.

**Figure 6:**
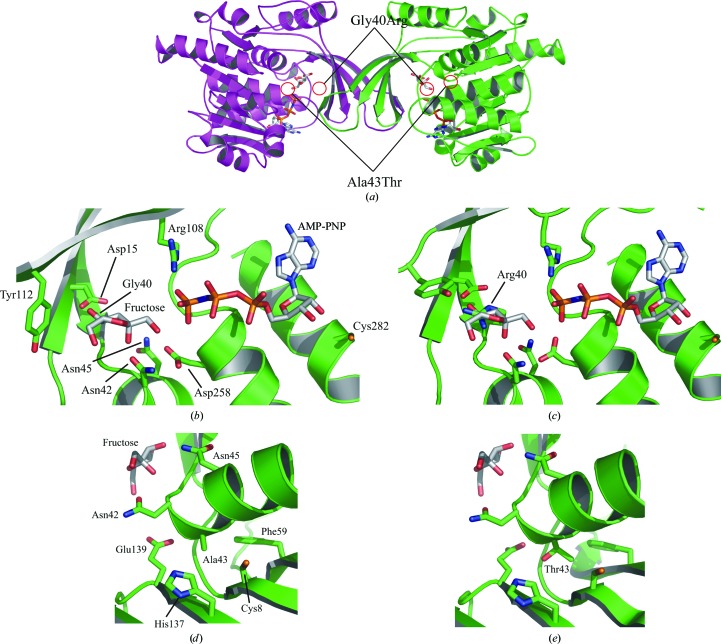
Energy-minimized models of the Gly40Arg and Ala43Thr mutants. (*a*) The locations of these residues in the KHK-A structure are highlighted in the ribbon representation. Stick and ribbon diagrams show unmodelled Gly40 (*b*) and Ala43 (*d*) and modelled Arg40 (*c*) and Thr43 (*e*). In (*d*) and (*e*) the AMP-PNP has been removed for clarity. These figures were generated using *PyMOL* (DeLano, 2002 ▶).

The Gly40Arg mutation renders both the KHK-A and KHK-C isoforms inactive and largely insoluble when expressed in *E. coli* (Asipu *et al.*, 2003 ▶). It might therefore be expected to have a large effect on the structure of KHK *in vivo*. Gly40 lies at the end of β3b and forms part of the binding pocket for fructose (Fig. 6 ▶). It is the first glycine of the highly conserved diglycine repeat (Wu *et al.*, 1991 ▶; Bork *et al.*, 1993 ▶) that links β3 and α1 and packs parallel to the fructose ring. The large arginine side chain would disrupt the diglycine-repeat motif and possibly prevent the formation of the closed conformation. The energy-minimized structure of Gly40Arg revealed that minor rearrangement of the highly conserved Asp15 on β2 is required to avoid any steric clashes. The model predicts changes in the local hydrogen-bonding network with three additional hydrogen bonds (Arg40 N^∊^—Asp15 O^δ1^, Arg40 N^η1^—Asp15 O^δ2^ and Arg40 N^η2^—Asn42 O^δ^) and the loss of one hydrogen bond (Asp15 O^δ2^—Gln38 N^∊2^). Superposition of the ternary-complex structure shows that the side chain of Arg40 would occupy the substrate site and obstruct fructose binding, explaining why this mutant renders both the KHK-A and the KHK-C isoforms inactive.

The Ala43Thr mutation has a much milder effect upon KHK enzyme activity, rendering the recombinant KHK enzymes more thermolabile than the wild type while having little impact on the kinetic parameters (Asipu *et al.*, 2003 ▶). Enzyme assays show a differential effect of the mutation in the two isoforms. Recombinant KHK-A is very stable up to 333 K and the Ala43Thr mutation reduces this thermostability by about 5 K (Asipu *et al.*, 2003 ▶). Recombinant KHK-C, on the other hand, is only stable to 313 K and since the Ala43Thr mutation lowers this by a further 5 K, this presumably results in a loss of enzyme activity at physiological temperature. The main predicted effect of Ala43Thr is therefore on KHK-C activity *in vivo*, which is consistent with the directly observed loss of activity in the liver. It is possible that KHK-A activity in peripheral tissues (*e.g.* brain, heart and smooth muscle) is relatively preserved, although this has not been directly determined, and if so the physiological importance of such a selective impact is unknown.

Although the Ala43Thr mutation results in the protein being less stable than the wild type, the protein is soluble and can be expressed in *E. coli*. However, crystallization trials using both a wide screen of commercially available conditions and the pre-determined conditions for KHK-A only resulted in very small needle clusters, so the mutation was modelled.

Ala43 lies in α1, which forms part of the substrate-binding pocket, and the neighbouring residues Asn42 and Asn45 make direct hydrogen bonds to fructose (Fig. 6 ▶). The side chain of Ala43 points away from the substrate site towards the interior of the protein and packs closely with the surrounding residues. The model predicts steric clashes between the Thr43 side chain and Glu139 C^β^ and C^α^ of the highly conserved Gly10 in the central β-sheet. To accommodate the mutation, local structural rearrangements are required, although the energy-minimized model showed only minor changes. Although these changes are small, the bulky threonine side chain would disrupt the packing in the core of the main domain and may possibly be responsible for the loss of thermostability.

## Supplementary Material

PDB reference: wild-type KHK-A, 2hqq, r2hqqsf

PDB reference: KHK-A cocrystallized with AMP-PNP, 2hw1, r2hw1sf

PDB reference: wild-type KHK-C, 3b3l, r3b3lsf

Supporting information file. DOI: 10.1107/S0907444908041115/be5117sup1.pdf

## Figures and Tables

**Table 1:** Crystallographic data-collection and refinement statistics Values in parentheses are for the outermost resolution shell.

	KHK-APtCl_6_	KHK-A	KHK-AAMP-PNPfructose	KHK-C
Crystallization conditions	0.50.9*M* ammonium sulfate, 0.5*M* lithium sulfate, 0.1*M* sodium citrate pH 5.46.0	As for KHK-APtCl_6_	As for KHK-APtCl_6_. Transfer buffer: 10% PEG 400, 0.1*M* Tris pH 8.5, 10m*M* AMP-PNP, 8m*M* fructose, 6m*M* Mg^2+^	1822%(*w*/*v*) PEG 3000, 0.1*M* sodium acetate pH 4.04.5
Space group	*I*222	*I*222	*I*222	*P*2_1_2_1_2_1_
Unit-cell parameters				
*a* ()	61.4	61.3	61.3	90.6
*b* ()	109.2	109.6	107.8	140.7
*c* ()	146.5	146.3	146.7	179.3
Resolution ()	2.8	1.86	2.1	2.90
Molecules per ASU	1	1	1	4
Observed reflections	44160	297643	105516	204215
Unique reflections	12313	41488	27884	51375
Completeness (%)	98.7 (96.3)	99.5 (99.2)	97.2 (91.2)	99.7 (99.9)
*I*/(*I*)	13.5 (3.2)	6.5 (2.4)	8.0 (2.0)	13.5 (6.8)
*R*_merge_ † (%)	5.4 (24.1)	6.4 (32.0)	6.8 (34.3)	6.0 (35.4)
Heavy-atom analysis				
Resolution range ()	10.03.0			
Mean figure of merit	0.42			
No. of reflections phased	9907			
No. of sites	1			
*R* factor (%)		20.5	19.2	23.6
*R*_free_ ‡ (%)		23.4	22.8	28.1
No. of protein atoms		2277	2277	8894
No. of solvent molecules		268	249	18
No. of ligand atoms		0	43	0
No. of sulfate atoms		15	5	0
Average overall *B* factor (^2^)		30.6	29.7	81.2
R.m.s.d. bond lengths§ ()		0.013	0.013	0.013
R.m.s.d. bond angles§ ()		1.3	1.3	1.4
Ramachandran analysis¶ (%)				
Most favoured regions		99.0	98.6	95.9
Outliers		0	0	0

† *R*_merge_ = 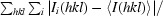 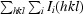.

‡ *R*_free_ was calculated with 5% of reflections set aside randomly.

§ Based on the ideal geometry values of Engh Huber (1991 ▶).

¶ Ramachandran analysis using *MolProbity* (Lovell *et al.*, 2003 ▶).

## Footnotes

1 Supplementary material has been deposited in the IUCr electronic archive (Reference: BE5117). Services for accessing this material are described at the back of the journal.
