# Supplementary material for: Structures of alternatively spliced isoforms of human ketohexokinase
Source: Acta Crystallogr D Biol Crystallogr. 2009 Feb 20;65(Pt 3):201–11. doi: 10.1107/S0907444908041115 (PMC2651755; doi:10.1107/S0907444908041115)

Supplementary figure. Comparison between human KHK-A and KHK-C. Ribbon diagrams of KHK-A in the presence and absence of fructose + AMPPNP, KHK-C subunits A and B. For all structures, the subunit is shown in the same orientation. The open and closed states of the KHK-C subunits can be compared with the superpositioning of KHK-C subunits A and B using 129 C_{α} atoms of the central α/β fold. The alternative splicing of the KHK gene results in a different sequence for a single region of the chain between the two isoforms (residues 72 to 115), shown in red. The figures were generated using PyMol (DeLano, 2002).

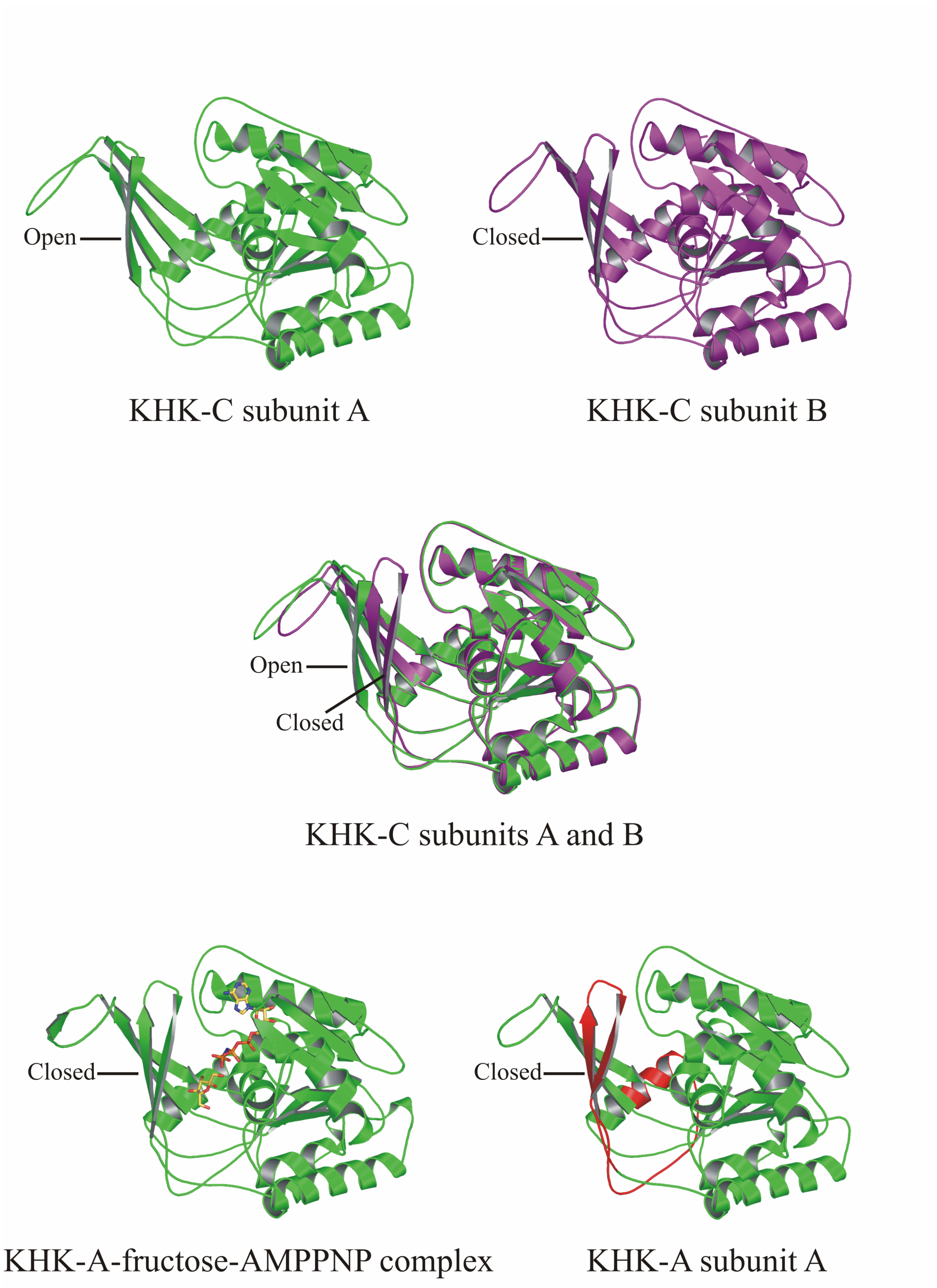

Supplement: Supplementary file 1 [file d-65-00201-sup1.pdf]
